# Geraniol attenuates oxidative stress and neuroinflammation-mediated cognitive impairment in D galactose-induced mouse aging model

**DOI:** 10.18632/aging.205677

**Published:** 2024-03-20

**Authors:** Peramaiyan Rajendran, Fatma J. Al-Saeedi, Rebai Ben Ammar, Basem M. Abdallah, Enas M. Ali, Najla Khaled Al Abdulsalam, Sujatha Tejavat, Duaa Althumairy, Vishnu Priya Veeraraghavan, Sarah Abdulaziz Alamer, Gamal M. Bekhet, Emad A. Ahmed

**Affiliations:** 1 Department of Biological Sciences, College of Science, King Faisal University, Al-Ahsa 31982, Saudi Arabia; 2 Department of Nuclear Medicine, College of Medicine, Kuwait University, Safat 13110, Kuwait; 3 Laboratory of Aromatic and Medicinal Plants, Center of Biotechnology of Borj-Cedria, Technopole of Borj-Cedria PBOX 901, Hammam-Lif 2050, Tunisia; 4 Department of Biomedical Sciences, College of Medicine, King Faisal University, Al-Ahsa 31982, Saudi Arabia; 5 Department of Biochemistry, Saveetha Dental College and Hospitals, Saveetha Institute of Medical and Technical Sciences, Chennai 600077, Tamil Nadu, India; 6 Department of Zoology, Faculty of Science, Alexandria University Egypt, Alexandria 21544, Egypt; 7 Laboratory of Molecular Physiology, Zoology Department, Faculty of Science, Assiut University, Assiut 71515, Egypt

**Keywords:** D-galactose, geraniol, cognitive disorder, Nrf2, ^99m^Tc-HMPAO, apoptosis

## Abstract

D-galactose (D-gal) administration was proven to induce cognitive impairment and aging in rodents’ models. Geraniol (GNL) belongs to the acyclic isoprenoid monoterpenes. GNL reduces inflammation by changing important signaling pathways and cytokines, and thus it is plausible to be used as a medicine for treating disorders linked to inflammation. Herein, we examined the therapeutic effects of GNL on D-gal-induced oxidative stress and neuroinflammation-mediated memory loss in mice. The study was conducted using six groups of mice (6 mice per group). The first group received normal saline, then D-gal (150 mg/wt) dissolved in normal saline solution (0.9%, w/v) was given orally for 9 weeks to the second group. In the III group, from the second week until the 10th week, mice were treated orally (without anesthesia) with D-gal (150 mg/kg body wt) and GNL weekly twice (40 mg/kg body wt) four hours later. Mice in Group IV were treated with GNL from the second week up until the end of the experiment. For comparison of young versus elderly mice, 4 month old (Group V) and 16-month-old (Group VI) control mice were used. We evaluated the changes in antioxidant levels, PI3K/Akt levels, and Nrf2 levels. We also examined how D-gal and GNL treated pathological aging changes. Administration of GNL induced a significant increase in spatial learning and memory with spontaneously altered behavior. Enhancing anti-oxidant and anti-inflammatory effects and activating PI3K/Akt were the mechanisms that mediated this effect. Further, GNL treatment upregulated Nrf2 and HO-1 to reduce oxidative stress and apoptosis. This was confirmed using ^99m^Tc-HMPAO brain flow gamma bioassays. Thus, our data suggested GNL as a promising agent for treating neuroinflammation-induced cognitive impairment.

## INTRODUCTION

Chronic oxidative stress is a major factor in neurodegenerative diseases and aging due to the accumulation of reactive oxygen species [1]. Aging is a natural phenomenon associated with an increase in reactive oxygen species and then a dysfunction in the antioxidant defense system, leading to a higher risk of diseases such as Alzheimer’s, cancer and heart diseases [2]. The decline of brain function and cognitive impairment by aging attribute to oxidative stress, DNA damage and then mutations induction, which cause abnormal protein aggregation, neuroinflammation, and neurodegeneration [3–5].

Several recent studies have clarified the role of D-gal injection in inducing brain aging through increasing oxidative damage, inflammation and apoptosis, as well as lowering brain function leading to cognitive impairment [6–9]. In fact, our body can synthesize D-gal, which is a reducing sugar that constitutes a part of glycolipids and glycoproteins, obtained from a variety of foods. The accumulation of D-gal *in vivo* may result in the formation of advanced glycation end products (AGEs). A class of chemicals known as AGEs created primarily through reducing sugars glycation (non-enzymatic) with amino acids, proteins, and lipids. Through pro-inflammatory effects and their specific harmful destruction of cells and via binding to certain receptors notably RAGE, AGEs accumulate in diseases related to the ageing process and are connected with significant roles in ageing. RAGE is found on many different cell membranes, including those of inflammatory cells and neurons [10]. The connection between AGEs and RAGE promotes the development of neuroinflammation by stimulating microglia and the associated NF-kB pathway. D-gal induced aging showed to develop neuroinflammation via AGEs/RAGE/NF-κB axis along with the elevation of cytokines level such as IL-6, TNF-α, and IL-1β. There by it increases the aging associated neuroinflammation via D-gal [11]. D-gal-treated mice showed increasing in serum AGE levels, memory delay time, and skin hydroxyproline concentration. Additionally, D-gal-treated mice displayed a marked reduction in lymphocyte mutagenesis, motor activity, the synthesis of IL-2, and the activity of the superoxide dismutase (SOD) enzyme [12]. D-gal can trigger free radical production, which results in oxidative stress via MDA generation with increasing level of lipid peroxidation and then apoptosis [8, 9] D-gal is capable of undergoing auto-oxidation processes in the presence of molecular oxygen. This process leads to the generation of superoxide radicals (O_2_·-) and hydrogen peroxide (H_2_O_2_), which are classified as reactive oxygen species (ROS). Subsequently, these ROS might generate additional reactive species, hence contributing to the occurrence of oxidative stress within cellular structures. The auto-oxidation of D-gal is a non-enzymatic chemical reaction that directly produces ROS [13, 14]. This oxidative stress is particularly relevant in the context of neurodegenerative disorders, as it has been associated with the pathogenesis and progression of conditions such as Alzheimer’s and Parkinson’s disease [15]. The delivery of D-gal in the indirect pathway is reported to affect multiple biological systems, resulting in heightened oxidative stress. D-gal has a negative impact on mitochondrial function. Mitochondria play a crucial role as cellular organelles in generating energy, and any malfunction in these organelles might result in an elevated production of ROS. Furthermore, the administration of D-gal showed to be associated with disruptions in the balance of metal ions in the body, namely iron. This can lead to the production of ROS through Fenton reactions. In summary, the oxidation of D-gal can occur through direct and indirect pathways, which include different physiological processes. These pathways can generate ROS, resulting in oxidative stress and potential harm to cellular components. It is crucial to acknowledge that the precise mechanisms may differ based on the particular experimental settings and the biological system being studied [16]. On the other hand, cells have protective mechanisms and antioxidant system protects cells from oxidative and proteotoxic stress. D-gal was found to increase the aging at earlier stage by inducing the function of Band 3 protein (B3p), it alters the antioxidant system and Hb, it increases the Hb glycation and membrane level oxidative stress. Thus, B3p would be proper target molecule for the balancing of oxidative stress and antioxidant level during aging [17]. One of these molecules is the nuclear factor (erythroid-derived-2)-like 2 (Nrf2). The antioxidant response element (ARE) is responsible for the activation of Nrf2. The function of Nrf2 is to protect cells from being damaged by oxidative stress induced by reactive oxygen or nitrogen species [18–20].

One of the complex brain structures that has a major role in learning and memory is the Hippocampus. Studies reported that the hippocampus-dependent object-place recognition task is associated with long-term spatial memory is impaired in aged mice and also in mice exposed to D-gal [21–23]. In brain, Nrf2 and related signaling pathways help to combat oxidative stress, making them appealing targets in treating cognitive decline. In this regard, several recent studies have documented that with aging, the decline in cognitive abilities is associated with a decrease in Nrf2 expression level. Therefore, Nrf2 pathway is a promising target for treating neurodegenerative diseases [24–27]. Nrf2 signaling regulated by complex mechanisms at many levels, including transcription, post-translational modification and protein-protein interactions. Numerous mechanisms were involved in controlling the intracellular distribution, stability, and activity of Nrf2. Protein kinase B (Akt) and phosphoinositide 3 kinase (PI3K), are essential for modulating Nrf2 activity [28]. The PI3K/Akt pathway is activated, followed by nuclear translocation of Nrf2 and antioxidant enzyme induction during normal physiological condition [29]. On the other hand, D-gal-induced aging was reported to inhibit the PI3K/Akt pathway and inactivate Nrf2-mediated antioxidants expression in liver and brain [30, 31]. GNL is one of the acyclic isoprenoid monoterpenes, which can be extracted from the aromatic plants’ essential oils such as *Cinnamomum tenuipilum, Valeriana officinalis,* and other plants [32]. Researchers found that GNL has a variety of pharmacological effects, including, anti-inflammatory, antitumor, antioxidative and antimicrobial activities [33–35].

In the present study, GNL was tested for its effects on oxidative stress and neuroinflammation mediated cognitive impairment in D-gal-induced aged mice model. Mechanistic action of GNL on Nrf2 and AKT signaling pathways were explored. Biochemical and histopathological investigations conducted to understand the neuroprotective effect of GNL on hippocampus-of aged model and old mice relative to control. In addition, we performed behavioral analysis (Videos are Supplemented) and studied the spatial learning and memory loss in aged model and GNL treated aged model relative to control and old mice.

## MATERIALS AND METHODS

### Chemicals

Geraniol and D-gal were purchased from Sigma Aldrich in Germany. Antibodies against HO-1 (# PA5-77833), PARP (PA5-16452), pAkt (# 44-621G), NQO1 (# PA5-82294), pPI3K (#PA5-104853), BCL2 (# PA5-27094), PI3K (# PA5-29220), Nrf2 (# PA5-105664), β-actin (# PA5-78716) and Akt (# 44-609G) were obtained from Invitrogen; Thermo Fisher Scientific, Inc., (Waltham, MA, USA). Anti-cleaved caspase-3 (ab32042) antibody was purchased from Abcam (Branford, CT, USA). MDA (Cat no: 700870), SOD (Cat. no: 706002), GPx (Cat. no: 703102), and CAT (Cat. no: 707002) were measured with kits (from Cayman Chemical, Ann Arbor, MI, USA) and all experiments were done according to the manufacturer’s protocol.

### Animals and treatments

Thirty-six male albino mice, including 24 mice at 6 weeks of age, 6 mice at 4 months of age, and 6 mice at 16 months of age, were obtained from Charles River Laboratories (Écully, France). The mice weighed between 25 and 30, weighing 25–30 g. All methods carried out in accordance with relevant guidelines and according to regulations of King Faisal University. All experimental protocols were reviewed and approved by King Faisal University Research Ethics Committee (KFU-REC/2021-01-15) and then all before starting experiments. Mice were kept at room temperature of 22 ± 2°C with a 12/12 h light/dark cycle. Six groups (6 mice each per group) were randomly divided. While mice of the control group received equal amounts of physiological saline once daily for 9 weeks, the 2nd group (D-gal control, II) was given orally D-gal (150 mg/kg body wt) [36] dissolved in normal saline solution (0.9%, w/v) for 9 weeks. Mice of the third group (III) were treated weekly twice with GNL (40 mg/kg body wt) [37] alone in the first week. Starting from the second week and continuing through the 10th week, mice were treated with oral gavage (without anesthesia) using D-gal (150 mg/kg body wt), followed by GNL (40 mg/kg body wt) 4 hours later. Mice of the fourth group (the drug control group, IV), were treated with GNL from the second week up till the end of the experiment. In addition, 6 mice were used in each of two untreated groups at 4 months (group V) and 16 months (group VI) of age to analyze the cognitive impairment differences between mature and elderly mice. This was also done to determine whether treatment prevented or reversed the kinetics of cognitive decline. Every two days the water level was checked and changed. All methods were done in accordance with the guidelines and the ethical rules of King Faisal University. The mice were administered a 3% isoflurane to anesthetic and euthanized at the end of the experiment. To measure pro-inflammatory cytokines and biochemical markers, blood was collected under anesthesia condition (3% isoflurane) via cardiac puncture and serum was separated and stored at −80°C. Using a sensitive balance (Nimbus, MK, UK), brains, livers and spleen were excised and weighed before being fixed for histopathological analysis or homogenized for biochemical investigations.

### Behavioral analysis

We used different tests, including Morris’ water maze test, Y-maze test, object-place recognition, and open-field test, to study the behavior of control and treated mice (*n* = 6/group).

### Morris’ water maze test

Morris’ water maze test was used to estimate the spatial learning and memory after GNL and D-gal treatment. A circular tank having a diameter of 100 centimeters and a height of 20 centimeters was used. A white ink was added to the water (23 ± 1°C) to make the tank opaque. Placed the escape platform in the center of the pool. MWM entailed a navigation training trial and a probe test [38]. Each mouse subjected to four tests per day for 4 days straight as part of the learning and memory training. Whenever the mice couldn’t find the platform in 40 seconds, they were artificially guided to it and stayed there for 15 seconds to memorize it. The probe test without the platform was conducted for 45 seconds in the tank after the training trial.

### Y-Maze test

Studies have shown that excessive spontaneous alteration behavior is linked to enhanced cognitive performance. We followed the Buried Food Test, and the protocol was published by Mu Yang and Jacqueline N. Crawley in Simple Behavioral Assessment of Mouse Olfaction (CurrProtocNeurosci. 2009) [39]. A Y-maze was used to analyze mice’s behavior. It was made of brown pointed sheets with three arms that were 60 cm long, 15 cm high, and 15 cm wide at the bottom and top. Mouse sessions lasted from three to five minutes. In the right arms of Y, there is food. The mouse capability to remember if food is located in one arm or the other [40]. By this way conditions could affect learning and memory and help to analyze the mice behavior. For the food identification, each mouse’s arm position was recorded manually 3 times in different days for 5 minutes each.

### Open field test (object place recognition)

Based on previous studies, we modified a standard object recognition task to test the recognition of short-term object–place [39, 41]. Through the experiment, each mouse was allowed to adapt for 10 min in an open box (120 × 80 cm). Then they began training at 30-min intervals. Each mouse was given 5 minutes to practice object–place recognition in a quadratic box (150 × 100 cm W, 25 cm H). In the training session, two identical plastic objects were positioned diagonally 120 cm from the wall in the apparatus. Between the training and testing, there was a one-day rest period. Two identical objects were placed 120 cm from the wall and the mice during the test. Animals’ behavior was evaluated by how much time they spent looking at each object.

### Cytokine measurements

Serum samples were warmed to room temperature. ELISA kits were used to measure TNF-α (pg/mL), IL-1β (pg/mL), and IL-6 (pg/mL) in a serum sample, following the manufacturer’s protocol. Values are expressed as pg/mg of total protein. (Cat. no. BMS607-3 for TNF-α; BMS6002 IL-β1; Cat. no. 88-7064-77 for IL-6) obtained from Thermo Fisher Scientific (USA).

### Protein extraction from brain hippocampus

Isolation of hippocampus tissue (See Supplementary Methods) and extracts were lysed in lysis buffer (Tris-HCl (pH 7.4) - 50 mM, NaCl - 150 mM, EDTA - 1 mM, Triton X-100 0.1% (v/v), Sodium deoxycholate - 0.5% (w/v) and 0.01 mg·mL−1 aprotinin, 0.005 mg·mL−1 leupeptin, 0.4 mM PMSF and 4 mM NaVO4). Lysates were centrifuged at 13,000 rpm at 4°C for 25 minutes with a cocktail of phosphatase and protease inhibitors (Halt™ Protease Inhibitor Cocktail (Cat no: 78430), from Thermo FisherScientific™). Supernatants of all samples were stored at −80°C to be used for biochemical investigations. Protein concentration was determined using Bio-Rad protein estimation kit (Bio-Rad, Hercules, CA, USA).

### Biochemical analysis

Spectrophotometry used to measure CAT (U/mg protein), SOD (U/mg protein), MDA (nmol/mg protein) and GPX (U/mg protein) in the serum and hippocampus tissue. MDA, SOD, CAT and Gpx assay kits (Cayman Chemical, Ann Arbor, MI, USA) were utilized for analysis [42, 43].

### AChE evaluation

The Acetylcholinesterase (AchE) Activity Assay Kit (E-BC-K174, Elabscience, USA) was used to detect impairment of the cholinergic system in hippocampus tissue. This kit operates on the principle that AchE facilitates the breakdown of acetylcholine into choline. The choline then reacts with dithio p-nitrobenzoic acid (DTNB) to produce 5-mercapto-nitrobenzoid acid (TNB). The absorption peak of TNB occurs at a wavelength of 412 nm. In this study, we evaluated the enzymatic activity of Acetylcholinesterase (AchE) by measuring the rate of absorbance increase at a wavelength of 412 nm. The experimental procedures were conducted in accordance with the instructions provided by the manufacturer.

### ^99m^Tc-HMPAO brain flow gamma camera imaging and processing

To study the brain blood flow, animals were anesthetized, injected intravenously with 111 MBq of ^99m^Tc-HMPAO, then imaged with a single-head gamma (γ) camera (Philips camera; Odyssey LX) equipped with a high-resolution parallel hole collimator connected to a Dell computer [44]. The matrix was 64 × 64 pixels, and the photo peak was focused at 140 keV with a symmetric 10% window. A zoom factor of 4 was applied during each acquisition time. Dynamic whole-body imaging was performed in two phases: (1) the vascular phase at 1 sec/frame for 3 min, followed by (2) the parenchymal phase at 1 min/frame for 60 min after the ^99m^Tc-HMPAO injection. To draw a region of interest (ROI), all brain imaging frame data were combined in one frame, then ROI was drawn around the brain (target, T), the whole-body region (WB), and the background area. The net ^99m^Tc-HMPAO uptake of the brain was calculated as the net target count, NTC = (T-BG)/(WB-BG), in each of the groups and was expressed as the mean standard deviation (mean ± SD). The ROIs were outlined with equal pixel sizes to reduce differences among the groups.

### Histopathological evaluations

We collected brain, liver and spleen from mice, fixed them in 4% paraformaldehyde. Samples were kept at 4°C for 4 h, soaked them overnight in 100 mM sodium phosphate buffer (containing 30% sucrose), and embedded them in paraffin. After that, sections were stained with haematoxylin and eosin (H&E) and toluidine blue staining to be investigated under microscope.

### Western blot analysis

RIPA lysis buffer was used to extract proteins from brain hippocampus tissue homogenates. Tissue homogenates were collected by centrifugation at 12,000 rpm for 30 min at 4°C. Using a Bio-Rad protein assay, and bovine serum albumin (BSA) as a reference standard. We isolated equal amounts of protein (40 μg) on 10% sodium dodecyl sulfate–polyacrylamide gel electrophoresis (SDS-PAGE) and then transferred it to PVDF membranes. Block it with 5% nonfat dry milk in Tris-buffered saline with Tween 20 (TBST, 150 mMNaCl, 20 mMTris-HCl, and 0.1% Tween 20) for 1 hour at room temperature. Then membrane was incubated with primary antibody (Supplementary Table 1) for 12 h, IgG labeled goat anti-rabbit IgG (1:2000) or anti-mouse IgG (1:4000) was used as a secondary antibody (1.5 h) after membranes were washed three times with TBST. The samples were examined with a LI-COR chemiluminescence imaging system (3600-00-C-Digit Blot Scanner). Graphs of the densitometric band intensities were generated and analyzed using LI-COR Biosciences Image Studio Lite software (Lincoln, NE, USA) normalized to the untreated control band, which was set to 1.

### Statistical analysis

The statistical differences between the treated groups, the old mice and the control group were estimated using Two-way analysis of variance followed by Tukey’s post hoc test analysis using GraphPad Prism. Results were expressed as the mean ± SD (no = 6), with *p* < 0.05, *p* < 0.0001 which was considered to be statistically significant.

### Data availability statement

The data that support the findings of this study are available from the corresponding author upon reasonable request. Some data may not be made available because of privacy or ethical restrictions.

## RESULTS

### Effect of GNL on body weight and organ index

**Figure 1:**
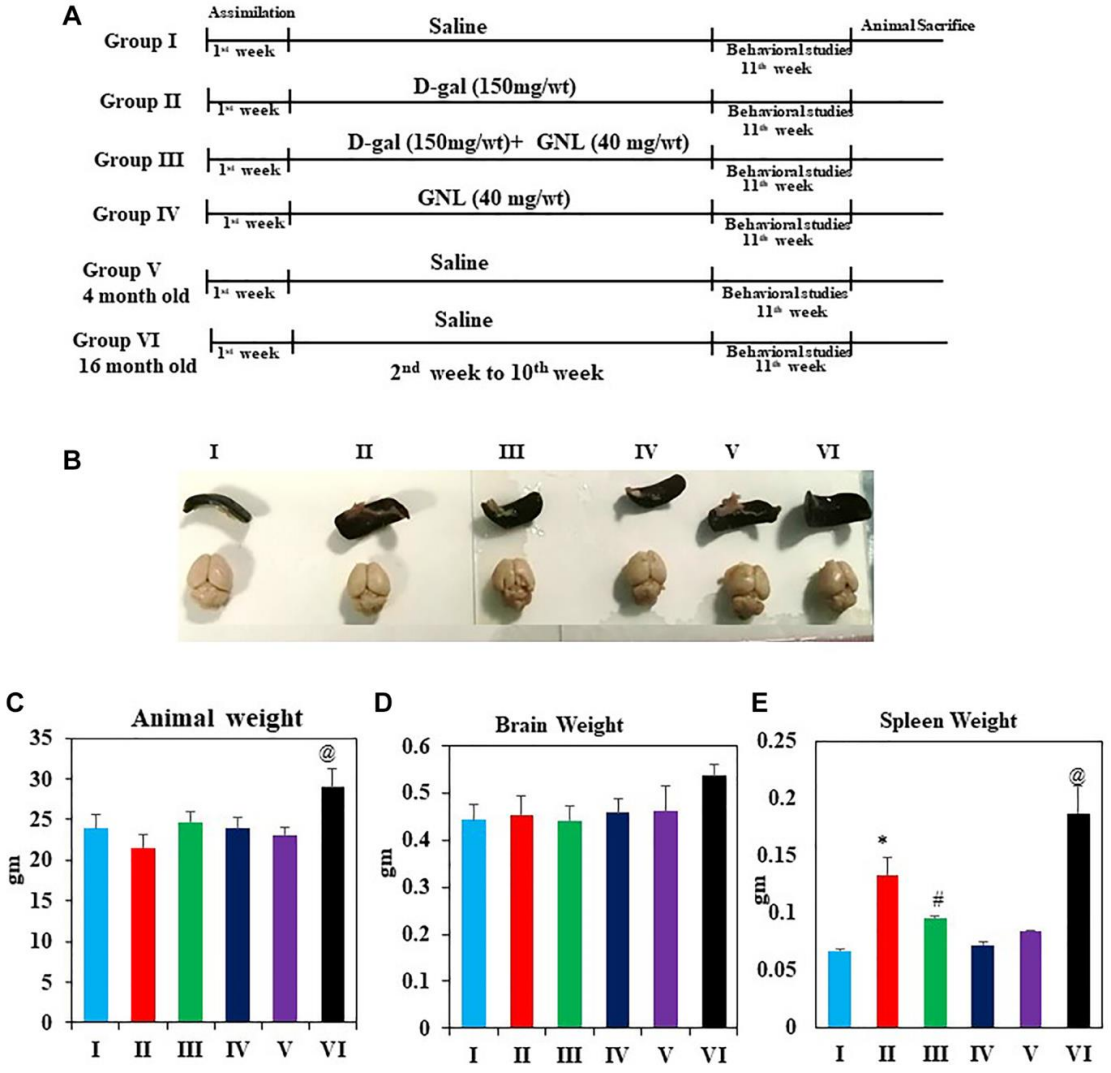
**Effect of GNL on body and organ weight.** (**A**) Illustration of the experimental procedure for D-gal and GNL in mice. The first group of animals was given physiological saline solution instead of D-gal (I), and the second group was given D-gal (150 mg/wt) dissolved in normal saline solution (0.9%, w/v) for 9 weeks (II). Animals in the third group of experiments were treated with GNL (40 mg/kg body wt) alone from the second to the 10th week by oral gavage (without anesthesia) with D-gal (150 mg/kg body wt), followed by GNL (40 mg/kg body wt) 4 h later (III). Animals in the fourth group were given GNL from the second week until the end of the experiment (IV). We included six mice from two untreated groups at 4 months (V) and 6 months (IV) of age for analysis of cognitive impairment. This was done to determine whether treatment prevented or reversed the kinetics of cognitive decline. (**B**) spleen and brain morphology (**C**) body weight (g), (**D**) brain weight (g), and (**E**) spleen weight (mg). Values are expressed as the mean ± SD (*n* = 6). ^*^P < 0.05 vs. control group, ^#^*P* < 0.05 vs. D-gal alone-treated mice. ^@^*P* < 0.05 young vs. old mice.

Figure 1A shows graphical abstract of the animal experimental. In Figure 1B after week 9 of treatment, the d-gal model group had a significant decrease on body weight relative to the control group (*p* < 0.05), however the other administrated groups reversed d-gal-induced weight loss (*p* < 0.05). In the meantime, brain index (Figure 1C) of the D-gal group did not significantly change as compared to the control group (*p* < 0.05). However, the administration of GNL (40 mg/wt) significantly attenuated the weight loss and increased spleen indexes in comparison to the D-gal group (Figure 1D, 1E). In group V and group VI, significant variations found in body weight and spleen, whereas no variation was detected in brain weight. Thus, GNL modulates the reduction of body weight and decreases brain and spleen indexes in D-gal model.

### Effect of GNL on mice behavior

#### Morris water maze

**Figure 2:**
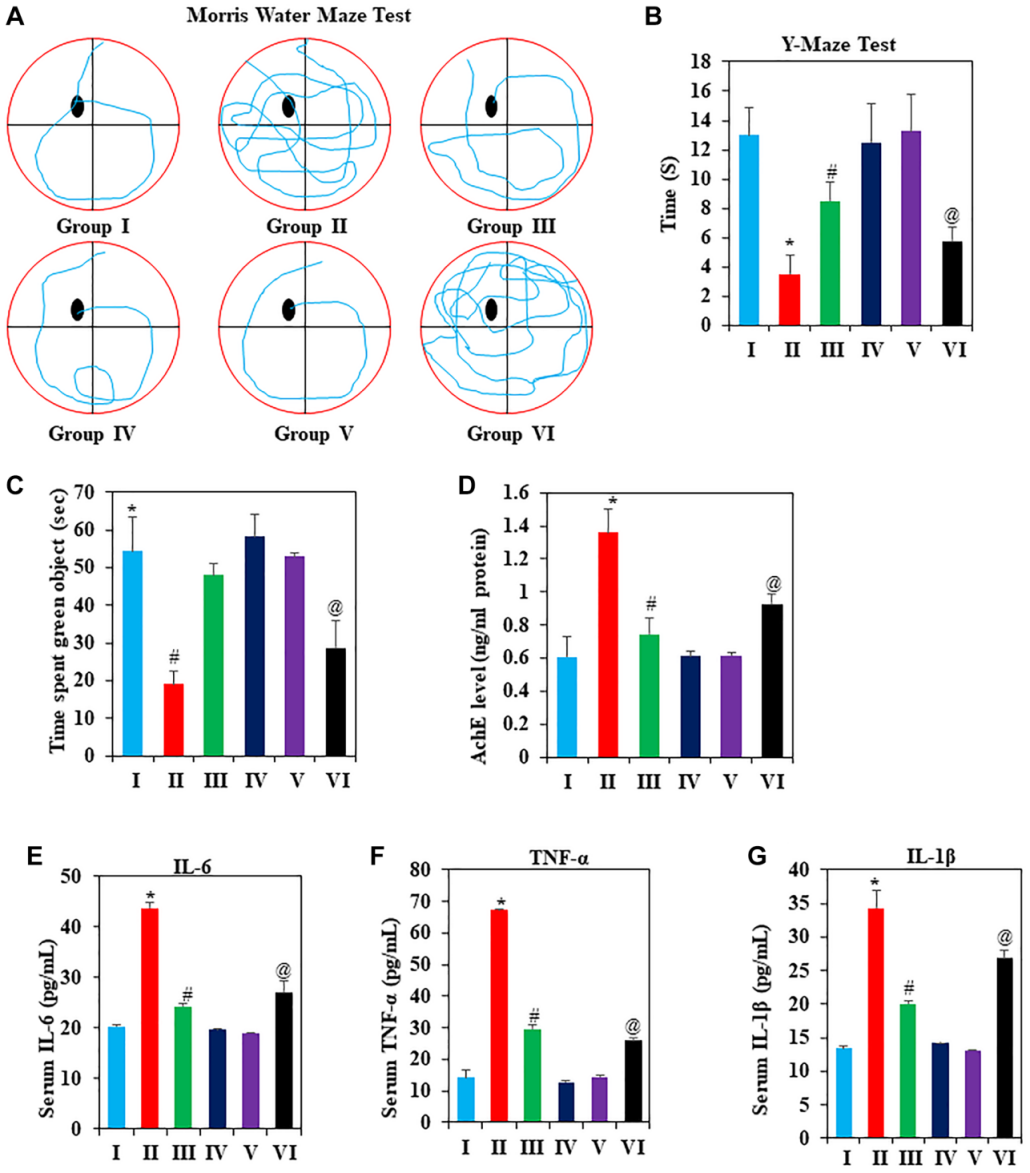
**The effects of GNL on cognitive decline induced by D-gal.** (**A**) Effect of GNL on Mouse Roadmap in the Morris Water Maze Test; (**B**) Effect of GNL on the Y-maze task, analyzed for spatial working memory in D-gal-induced mice. (**C**) Effect of GNL on object place recognition. (**D**) Effect of GNL on AchE level in D-gal-induced mice (ng/ml protein). Effect of GNL on inflammatory cytokines in serum, such as (**E**) IL-6 (pg/ml), (**F**) TNF-α (pg/ml), and (**G**) IL-1β (pg/ml). Group I: Control; Group II: D-gal alone (150 mg/wt); Group III: D-gal (150 mg/wt) with GNL (40 mg/wt); and Group IV: GNL alone (40 mg/wt). Group V: 4-month-old young animals; Group VI: 16-month-old. Values are expressed as the mean ± SD (*n* = 6). ^*^P < 0.05 vs. control group; ^#^*P* < 0.05 vs. D-gal alone-treated mice. ^@^*P* < 0.05 young vs. old mice.

The effects of GNL on spatial memory and learning were studied using the Morris water maze [45]. Chronic D-gal administration resulted in a significant longer time for mice to reach the platform (*P* < 0.05), while GNL therapy induced a significant reduction in escape latencies (Figure 2A) (Supplementary Video 1) (*P* < 0.05). On the other hand, D-gal-treated aging mice spent significantly less time in the goal area than the control group in the spatial probe trial (Supplementary Video 1) (*P* < 0.05). In these poor conditions, mice in the D-gal plus GNL group showed remarkable reversion. Additionally, the aging mimic mice in the model group displayed similar behavior to the group VI. On the other hand, aged animals have a longer latency to reach the platform compared with young animals under 4 months (Group IV). These findings demonstrated the effect of GNL to restore the D-gal-induced deficit in memory and learning abilities.

#### Y-maze task

Using the Y-maze task, we analyzed spatial working memory using spontaneous alteration. D-gal treated mice were less likely to enter food rewards, which placed around its arms. This indicates no exploratory behavior. A significant increase in spontaneously altered behavior was observed in mice, which received GNL either alone or in a combination with D-gal (*P* < 0.05), indicating that GNL improved memory in D-gal-treated mice (Figure 2B) (Supplementary Video 2). It doesn’t appear that there are any significant differences between groups III (Figure 2B) and control group in behavior to enter food rewards placed arms (Figure 2B). In addition, compared to 4-month-old young animals, animals in group VI were significantly less likely to enter the food rewards arm. Thus, GNL appears to attenuate D-gal-induced ageing in mice model.

#### GNL improves the D-gal-induced decline in object recognition

We explored the effect of GNL treatment on novel object recognition (NOR). When comparing the NOR test to the training session, a noticeable increase in object recognition index was observed in the control group. D-gal treatment also exacerbated the amnesic effect (Figure 2C) (Supplementary Video 3), while treatment with GNL (40 mg/kg body wt) (*p* < 0.05) ameliorated it (Supplementary Video 3). Long-term exposure to D-gal can lead to a recognition deficit. GNL could improve the memory impairment by D-gal treatment. The young animals in Group V moved quickly and used active recognition, while the old animals moved slowly, often stood still, and didn’t explore.

### Effects of GNL on acetylcholinesterase (AChE) activity in aged mice model

Acetylcholinesterase (AChE) affects learning and memory and controls cholinergic synapses and its activity, and considered as a reliable indicator of cholinergic activity [46]. As indicated in Figure 2D the AChE activity in the D-gal group is significantly higher than in the control group. GNL showed to reduce the levels of AChE in the D-gal-induced elderly mice group much more than in the control aging mice group. In group VI, the levels were significantly higher than those in group V. This proved that GNL can improve cognitive decline through modulating the level of AChE.

### Effect of GNL on pro-inflammatory cytokines induced by D-gal

The D-gal treated mice overexpress inflammatory cytokines, which is in line with previous reports [47, 48]. We found that pro-inflammatory cytokines such as TNF-α, IL-6, and IL-1β were two times higher in the D-gal-treated group than in the control group (Figure 2E–2G). Nevertheless, within the D-gal-treated group, serum levels of TNF-α, IL-6, and IL-1β significantly reduced as compared to the D-gal model group with GNL administration (*P* < 0.05). This indicates an anti-inflammatory role for GNL on D-gal induced immune defect in mice.

### Effect of GNL on antioxidants level and activities of MDA induced by D-gal

**Figure 3:**
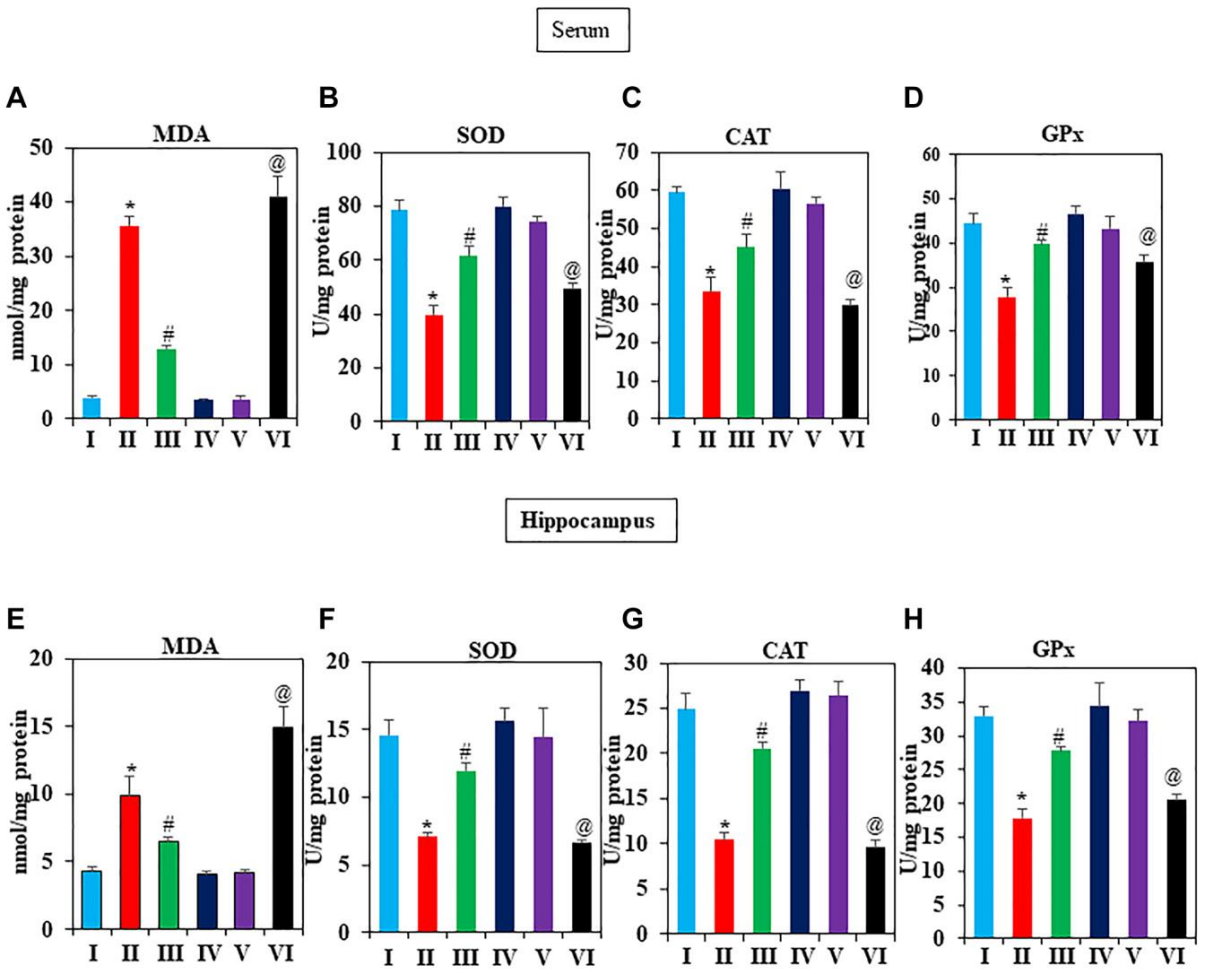
**Effect of GNL on antioxidant activity in D-gal-induced mice serum and hippocampus.** (**A**) serum, MDA level control, and treated animals (nmol/mg protein), (**B**) SOD (U/mg protein), (**C**) CAT (U/mg protein), and (**D**) GPx (U/mg protein). (**E**) Hippocampus MDA level control and treated animals (nmol/mg protein), (**F**) SOD (U/mg protein), (**G**) CAT (U/mg protein), and (**H**) GPx (U/mg protein). Values are expressed as the mean ± SD (*n* = 6). ^*^P < 0.05 vs. control group, ^#^*P* < 0.05 vs. D-gal alone-treated mice. ^@^*P* < 0.05 young vs. old mice.

Increasing evidence indicated that oxidative stress is connected to the biology of aging [49, 50]. Therefore, we investigated several antioxidants that could scavenge ROS in the serum and hippocampus of D-gal induced mice, including MDA, SOD, CAT, and GPX. According to Figure 3A–3D, there was a noticeable increase in MDA, and decreased SOD, CAT, and GPx levels after D-gal exposure compared to the vehicle group (*P* < 0.05). However, in serum GNL treatment reversed the decline due to its antioxidant activity. Group IV mice treated with GNL alone showed no significant difference in serum CAT, GPx, and SOD. On the other hand, the MDA levels in hippocampus homogenates significantly increased in the D-gal group, whereas GNL treatment at 40 mg/wt significantly abated the rise in hippocampus MDA levels (Figure 3E). GNL treatment enhanced antioxidant activity in the hippocampus, according to our results (Figure 3F–3H). In addition, as shown in Figure 3A, 3F, the MDA levels of aged mice (Group VI) were much higher than those of young control mice (V). Taken together, these data revealed a protective effect of GNL on D-gal induced oxidative stress in brain hippocampus and serum of mice.

### Effect of GNL on D-gal induced changes in the expression of PI3K/Akt

**Figure 4:**
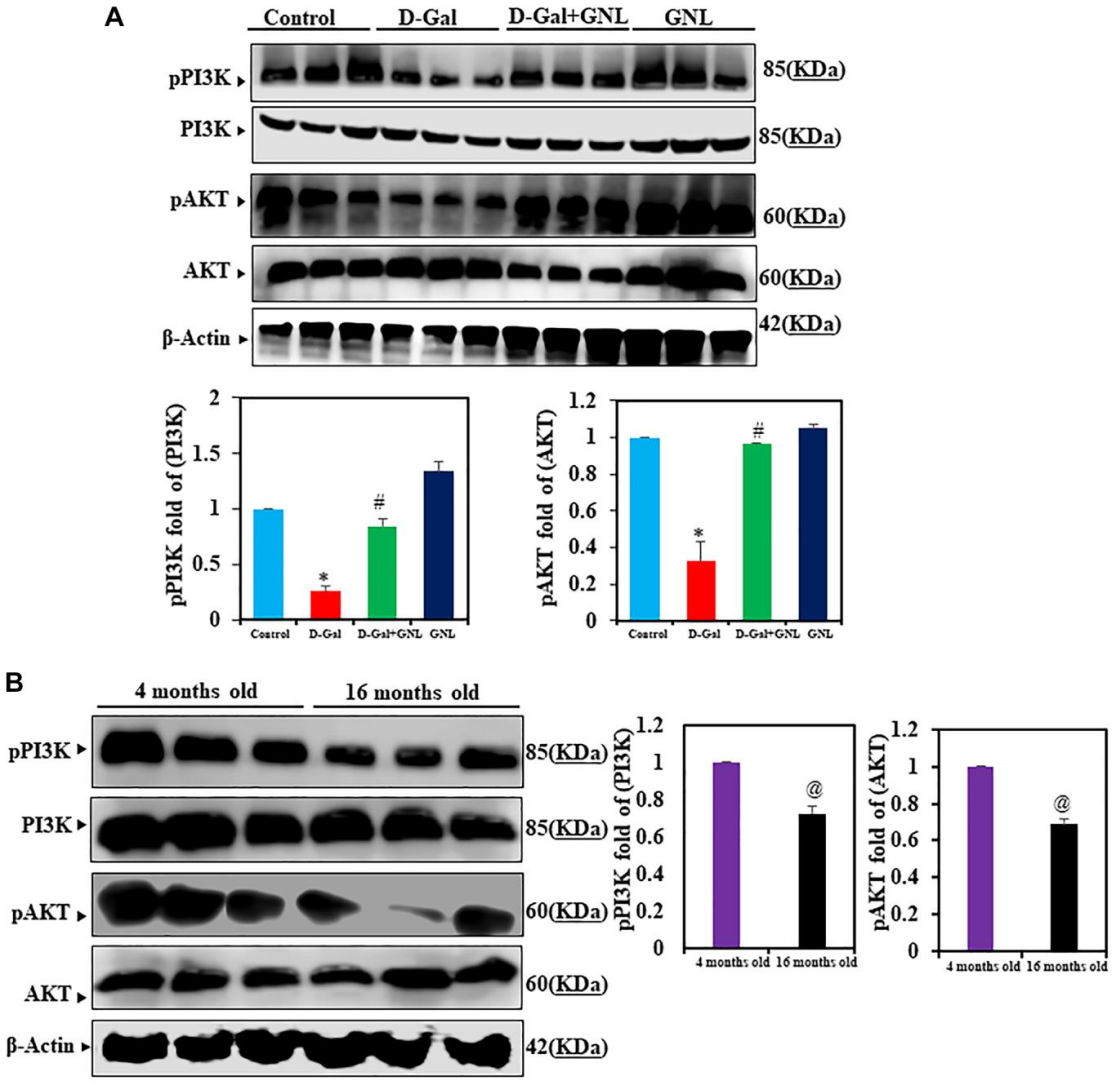
**GNL effects of PI3K/AKT signaling in the hippocampus of D-gal-induced mice.** (**A**) pPI3K and pAKT protein analysis by Western blot. Three independent experiments are shown here. SDS-PAGE resolved the protein from each sample, and Western blots were done. The internal load controllers were AKT and PI3K. Densitometry analysis calculated changes in protein bands as 1.0-fold, as shown below the gel. (**B**) 4- month-old young control and 16-month-old mice hippocampus tissue were analyzed for pPI3K and pAKT levels. The internal load controllers were AKT and PI3K. Densitometry analysis calculated changes in protein bands as 1.0-fold, as shown on the right side of the gel. Values are expressed as the mean ± SD (*n* = 6). ^*^P < 0.05 vs. control group; ^#^*P* < 0.05 vs. D-gal alone-treated mice. ^@^*P* < 0.05 young vs. old mice.

Next, we measured AKT and PI3K expression levels in control and treated groups. Although total PI3K and AKT protein levels were similar among all groups, D-gal exposure prevented PI3K signaling, as reflected by the reduced expression of pPI3K and pAKT expression. This inhibition of pPI3K and pAKT by D-gal was significantly alleviated by GNL treatment (Figure 4A). In 4-month-old animals (Figure 4B), the expression of PI3K and AKT showed no significant variation relative to group I, but in group VI (old mice), the level of phosphorylated of PI3K/AKT showed significant decrease.

### Effects of GNL on Nrf2, HO-1 and NQO-1 expressions in mouse hippocampus

**Figure 5:**
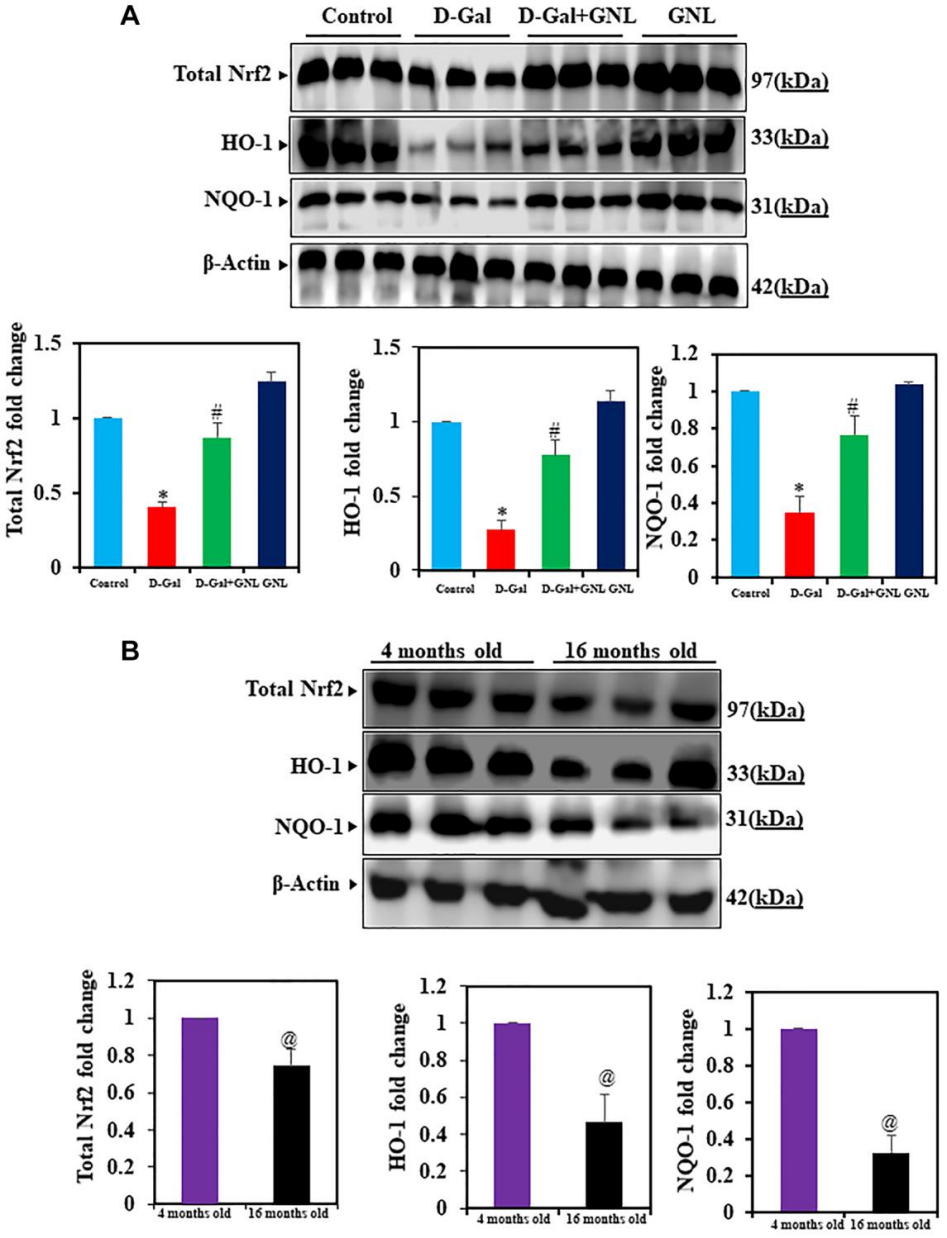
**Effect of GNL on D-gal-induced cognitive impairment mice of Nrf2, HO-1, and NQO-1 proteins in the hippocampus.** (**A**) The total Nrf2, HO-1, and NQO-1 protein levels were analyzed by Western blot. Three independent experiments are shown here. SDS-PAGE resolved the protein from each sample, and Western blots were done. The internal load controllers were β-actin. Densitometry analysis calculated changes in protein bands as 1.0-fold, as shown below the gel. (**B**) 4-month-old young control and 16-month-old mice hippocampus tissue were analyzed for total Nrf2, HO-1, and NQO-1. The internal load controllers were β-actin. Densitometry analysis calculated changes in protein bands as 1.0-fold, as shown on the right side of the gel. Values are expressed as the mean ± SD (*n* = 6). ^*^P < 0.05 vs. control group, ^#^*P* < 0.05 vs. D-gal alone-treated mice. ^@^*P* < 0.05 young vs. old mice.

As shown in Figure 5A, the expression of Nrf2, HO-1, and NQO-1 in GNL/D-gal treated mice were relative to the D-gal model mice. Further, Nrf2, HO-1, and NQO-1 expression in control aging mice (Figure 5A) decreased similarly to D-gal induced aging in mice. In group III (D-gal with GNL) treated mice activation of this protein was significantly elevated (*p* < 0.05). However, GNL alone, vehicle control and young (4 months old) weren’t significantly different (Figure 5B).

### GNL on expression of RAGE and BACE-1 proteins

**Figure 6:**
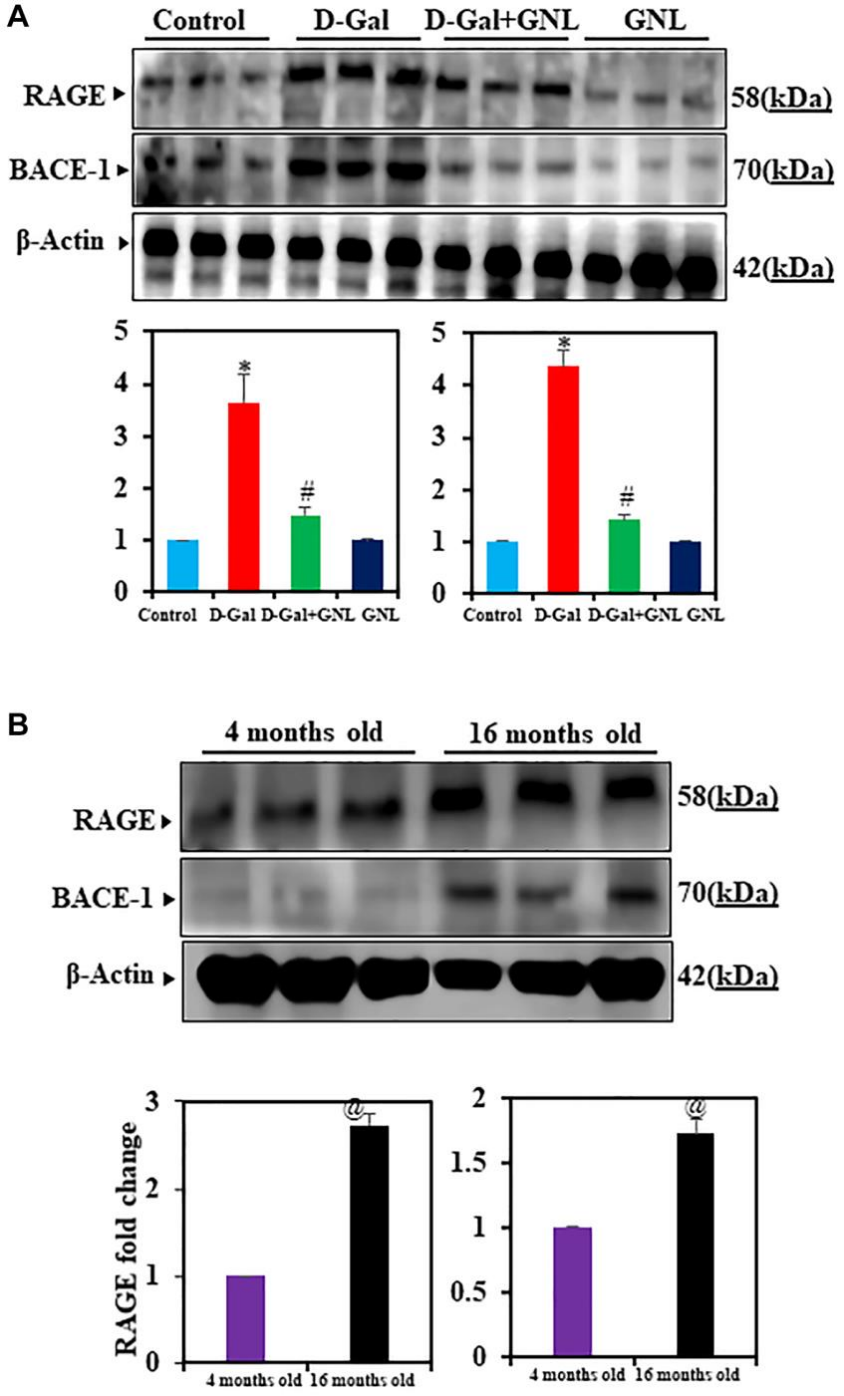
**Effect of GNL on D-gal-induced cognitive impairment mice of RAGE and BACE-1 proteins in the hippocampus.** (**A**) A Western blot analysis of the RAGE and BACE-1 protein levels. Three independent experiments are shown here. SDS-PAGE resolved the protein from each sample, and Western blots were done. The internal load controllers were β-actin. Densitometry analysis calculated changes in protein bands as 1.0-fold, as shown below the gel. (**B**) 4-month-old young control and 16-month-old mouse hippocampus tissue were analyzed for RAGE and BACE-1. The internal load controllers were β-actin. Densitometry analysis calculated changes in protein bands as 1.0-fold. Group I: Control; Group II: D-gal alone (150 mg/wt); Group III: D-gal (150 mg/wt) with GNL (40 mg/wt); and Group IV: GNL alone (40 mg/wt). Group V: 4-month-old young animals; Group VI: 16-month-old Values are expressed as the mean ± SD (*n* = 6). ^*^P < 0.05 vs. control group; ^#^*P* < 0.05 vs. D-gal alone-treated mice. ^@^*P* < 0.05 young vs. old mice.

The D-gal causes RAGE protein levels to rise, which stimulates hippocampus cells in neuro-inflammatory neuro disorders [51, 52]. To analyze GNL’s effect on RAGE and BACE-1, we did Western blots in all experimental groups. The immunoblots revealed a significant increase in RAGE and BACE-1 protein expression levels in the hippocampus regions of mice treated with D-gal alone compared to the control group. However, co-administration of GNL + D-gal markedly showed to reduce the expression of RAGE and BACE-1 (Figure 6A). Notably, the immunoblot results indicated that GNL was not toxic to normal mouse brains’ hippocampus, as no significant difference was observed between normal control mice and those treated with GNL alone (Figure 6A). RAGE and BACE-1 expression were also upregulated in control aging mice (16-month-old) (Figure 6B), similar to D-gal induced aging mice. In contrast, GNL alone, vehicle control and young (4 months old) had no effect (Figure 6B).

### GNL supplementation inhibited D-gal-induced hippocampal apoptosis

**Figure 7:**
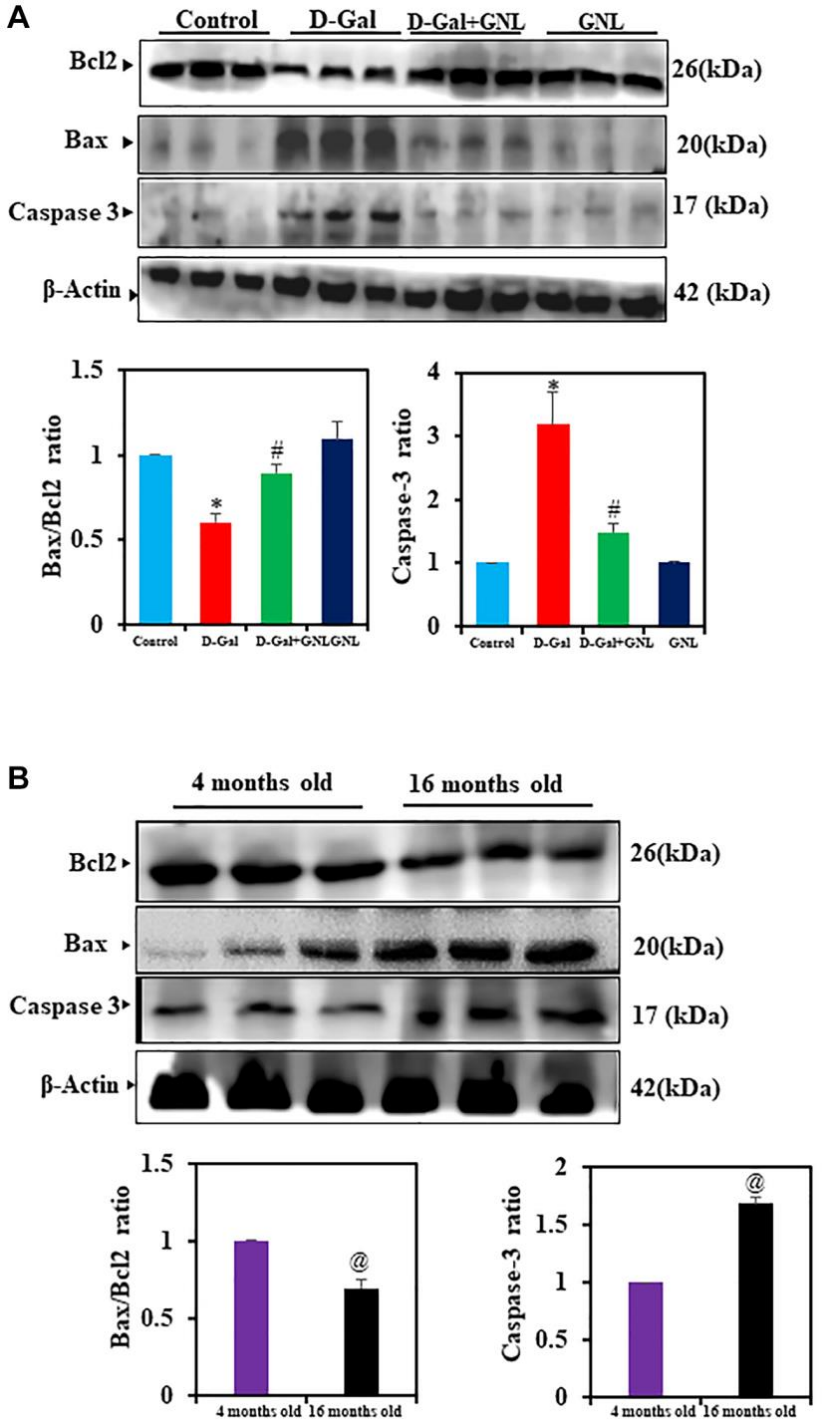
**The GNL attenuated excessive apoptosis in the mice’s hippocampus.** (**A**) The BCl2, BAX, and caspase-3 protein levels were analyzed by Western blot. Three independent experiments are shown here. SDS-PAGE resolved the protein from each sample, and Western blots were done. The internal load controllers were β-actin. Densitometry analysis calculated changes in protein bands as 1.0-fold, as shown below the gel. (**B**) 4-month-old young control and 16-month-old mice hippocampus tissue were analyzed for BCl2, BAX, and caspase-3. The internal load controllers were β-actin. Densitometry analysis calculated changes in protein bands as 1.0-fold, as shown below the gel. Values are expressed as the mean ± SD (*n* = 6). ^*^P < 0.05 vs. control group; ^#^*P* < 0.05 vs. D-gal alone-treated mice. ^@^*P* < 0.05 young vs. old mice.

D-gal induced aging was tied to hippocampal apoptosis, according to accumulating evidence [53, 54]. We used Western blot to examine whether, GNL supplementation inhibited apoptosis. Figure 7A, 7B show that the expression of caspase-3, as well as Bax/Bcl-2 ratios, were significantly increased by D-gal treatment compared to the control group. By supplementing GNL, the upregulation of these apoptosis-related proteins was dramatically attenuated. Control aging mice also showed an increase in the Bax/Bcl-2 ratio (Figure 7B). GNL alone, vehicle control and young (4 months old) had no effect (Figure 7A, 7B).

### GNL ameliorated histopathological alterations of the brain, liver and spleen

**Figure 8:**
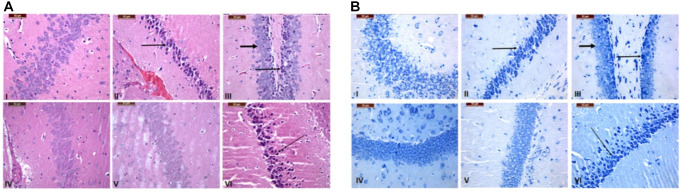
**Histopathological alteration in the brain tissue of the control and treated groups.** Hippocampus of regions stained with H&E (**A**) and Toluidine blue stain (**B**) (bar = 100 and 50 mm). Group I (6 weeks), the control group, showed a normal appearance and a high density of neurons with a light normal nuclear stain. Compared to the control group, Group II (6 weeks), which was made up of neurons that had dark nuclear staining (arrows) and focal disappearance, there was less density and necrosis in the D-gal group, which showed shrinking neurons with focal disappearance. In Group III (6 weeks), treatment with GNL revealed a protective effect, which showed mild neurodegeneration (thin arrows) with highly pronounced normal neurons (thick arrows). Normal appearance of neurons shown in Group IV (6 weeks) Treatment only. In Group V (4 months), a mild decrease in neuronal density has been shown compared to the control. However, severe neural loss, degeneration, and necrosis (arrows) were seen in old mice in Group VI (16 months) magnification 40X.

Hippocampus of the treated mice brain was stained with H&E (Figure 8A) and toluidine blue staining (Figure 8B) to investigate the histopathological changes of Cornu Ammonis (hippocampus proprius) of Hippocampus relative to the control. As depicted in Figure 8, D-gal induced neurodegeneration characterized by focal disappearance and a decrease in the density of the layers, along with necrosis represented by shrunken neurons with dark nuclear staining (arrows), compared to control group. In Group III (6 weeks), GNL exhibited a protective effect, wherein mild neurodegeneration was observed (thin arrows), along with highly pronounced normal neurons (thick arrow). Both control group and group IV (the drug control) showed normal appearance of neurons in the four- to five-layered Cornu Ammonis that was curvilinear and also the pyramidal layer harbors the pyramidal perikarya, which showed a massive density as. As shown in group IV (6 weeks). However, in-group V (4 months), mild decrease in neuronal density was similar to the control. On the other hand, severe neural loss, degeneration, and necrosis (arrows) were observed in old mice (16 months). The differences in cyto-architecture of the treated hippocampus versus control can be recognized even in old mice (16 months) as compared to the control. In consistent, D-gal induced histopathological alteration in liver and spleen (Supplementary Figures 1 and 2) and these changes modulated by GNL treatment. Taken together, these data support the protective effects of GNL at the histological level.

### Effect of GNL on ^99m^Tc-HMPAO brain flow gamma in D-gal induced aging mice

**Figure 9:**
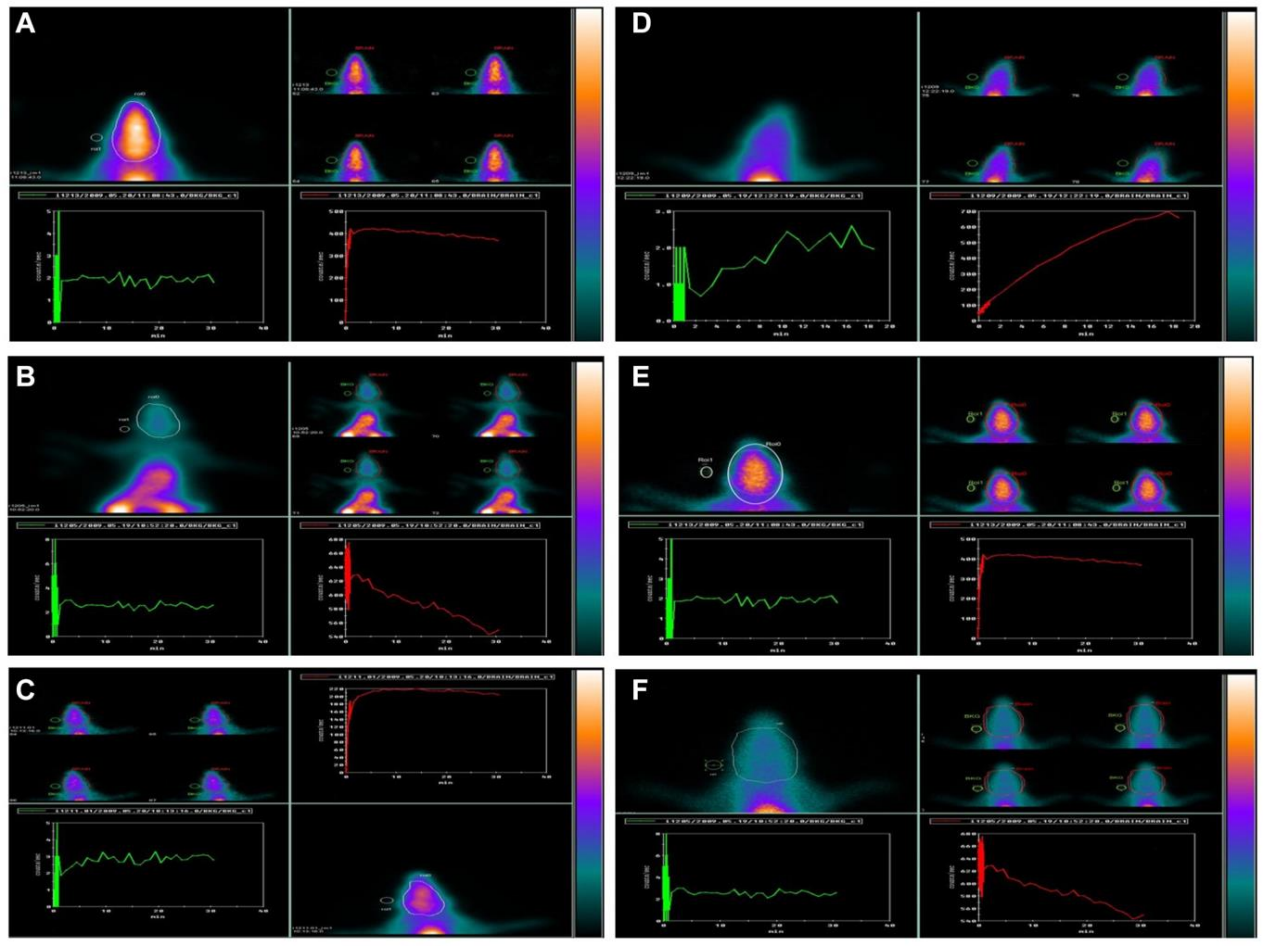
**Effect of GNL on D-gal-induced cognitive impairment mice by using ^99m^Tc-HMPAO (brain) animal imaging.** Composite 0–60-min images, time-activity curves, and regions of interest (ROIs) are shown: Group I: (**A**), Group II: (**B**), Group III: (**C**), Group IV: (**D**), Group V: (**E**), and Group VI: (**F**). Brain ROI. Abbreviations: BG: background; WB: whole body.

From a dynamic scan, a time-activity curve was created (Figure 9). The radioactivity concentration plotted against the time. This showed the concentration of ^99m^Tc-HMPAO within the animal’s brain, which was scanned and measured over 30-60 min. After intravenous injection, ^99m^Tc-HMPAO dynamic imaging using periods was steady for 30–60 min., then the uptake was determined. The frames were combined into one image. The ROIs were assigned to the brain, whole body, and background areas. The net target, ^99m^Tc-HMPAO brain, and whole body were normalized to background count, determined using the following formula: NTC = (T-BG)/(WB-BG), in each group, and expressed as the mean standard deviation (mean ± SD). The time-activity curve showed a similar increased pattern uptake for both the control, drug control, young animals, and treated group, group III, while group II and the aged group showed reduced uptake over the scanning time. The results of the unpaired multiple comparison tests recorded highly significant differences between the control, group II, and the aged one (*p* < 0:0001). Similarly, the analysis of variance, ANOVA test showed *p* < 0.0001, which reflects all the possible differences among all groups (Table 1).

**Table 1:** The net ^99m^Tc-HMPAO brain NTCs in experimental animals.

**Net ^99m^Tc-HMPAO brain NTCs**	**Mean ± SD**
**Control**	532.8 ± 1.5
**D-gal**	153.6 ± 1.3^*^
**D-gal + GNL**	512 ± 1.4
**GNL**	529 ± 1.2
**4 month young**	508 ± 1.5
**16 month old**	115.5 ± 1.3^*^

Data represent the mean ± SD of six independent experiments. ^***^p < 0.05 as compared to controls. ANOVA test showed *p* < 0.05.

## DISCUSSION

Life expectancy in the 21st century is rising, resulting in more age-related illnesses, such as memory impairment and Alzheimer’s disease. In this study, GNL was studied for its protective effect on D-gal-induced aging in mice. Interestingly, the D-gal induced aging model exhibits major similarities with the control aging model, demonstrating its potential for application in aging research [55]. The immune system plays a significant role in the course of aging. Ageing is closely related to the spleen, which is an important immune organ [56]. Based on the increased spleen index, it was evident that splenomegaly was present, and that immune function was downregulated, which resulted in an imbalance of cytokines in the body. In consistent, the current study showed that GNL could protect aging mice’s spleens. The function of liver gradually declines due to age-related structural atrophy and impairment [57]. Our study showed improvement in liver damage after GNL treatment. Those data suggested that concomitant GNL might be the best way to exert its anti-aging effects. GNL showed to improve spatial location learning and to reduce the extent of memory impairment (See Supplementary Videos 1–3) as well as to protect neurons in the hippocampus from oxidative damage and apoptosis induced by D-gal. Mechanistically, GNL appears to modulate the phosphorylation of PI3K/Akt and thus activates Nrf2, the key factor among PI3K/Akt downstream. Subsequently, GNL activates Nrf2 via the PI3K/AKT pathway to enhance the activity of antioxidant enzymes, like HO-1, NQO-1, and SOD.

The effect of GNL on memory impairment and learning in the D-gal-induced aging model is demonstrated here for the first time. In consistent with our results, GNL was reported to enhance a neuro-protective effect against ischemic injury in the brain and to pass efficiently through the blood–brain barrier [58, 59]. In the present study, we present robust evidence (Supplementary Videos 1–3) to show that GNL modulated the behavior changes induced in mice after D-gal treatment. In the Y-maze and open field test, we found significant variations between the vehicle and D-gal-treated mice, suggesting that D-gal injection causes motor abnormalities leading to impairments of novelty-induced exploratory behavior. Furthermore, our data show that GNL-treating for 9 weeks can reverse the D-gal-induced behavioral impairment. GNL was also shown to protect against D-gal-induced memory loss and spatial learning impairment. In consistent, recently, GNL has been suggested as a promising therapeutic agent in improving cognitive function and neurotoxicity induced in rats by ZnO-NPson, behavioral and biochemical evidence were provided [60].

Several age-associated characteristics have been reported to be related to the increase in reactive oxygen species, and thus the induction of oxidative stress and inflammation [61, 62]. Interestingly, in the current study, both aging models and old mice displayed these characteristics, including oxidative damage, apoptosis, and induction of an inflammatory response. The increase in ROS can cause NF-κB to move rapidly from the cytosol to the nucleus, resulting in an increase in inflammatory cytokine levels such as TNF-α, IL-6, and IL-1β.3. In this line, when we gave mice D-gal for 9 weeks orally, the serum concentrations of TNF-α, IL-1β, and IL-6 were markedly increased in the model animal. However, GNL substantially inhibited inflammatory cytokine production, suggesting its capability to attenuate the induced inflammatory response in D-gal-treated mice. In addition, D-gal attenuated antioxidant enzymes (SOD, CAT, and GPX) in the brain hippocampus and mice serum. The exhaustion of antioxidant enzymes is due to the increase in ROS production with aging, explaining the induction of age-related degenerative disease by D-gal [63]. However, the increase in brain hippocampus and serum SOD, CAT, and GPx activities in D-gal-treated mice is consistent with several previous studies that indicated the antioxidant properties of GNL. For instance, Lin and colleagues studied the effect of GNL on methicillin-resistant *Staphylococcus aureus* (MRSA) infections in mice, where GNL increased the antioxidant activity of SOD and reduced ROS and MDA in the kidneys [64]. In addition, Farokhcheh et al. showed the effect of GNL to reduce ROS in neurotoxicity cells in brain tissue [60]. As a result, this implies that the effect of GNL on aging could be mediated by modulating the imbalance between free radicals and antioxidants.

PI3K/Akt signaling is considered an important pathway for Nrf2 activation [65, 66]. Activation of the PI3K/Akt signaling pathway promotes the dissociation of downstream molecules, including Nrf2 and Keap1, thereby regulating the nuclear expression of Nrf2 [67, 68]. Several studies have hinted that the PI3K/Akt signaling pathway protects against D-gal-induced brain injury by modulating Nrf2 [68, 69]. Additionally, several natural compounds were found to improve the behavioral dysfunction and neurological deficits in D-gal-induced aging in mice via activation of the PI3K/Akt/Nrf2 pathway [70–72]. Results of the current work demonstrated that phosphorylated levels of PI3K and Akt are significantly upregulated after GNL treatment, which revealed that GNL can activate the PI3K/Akt signaling pathway to promote Nrf2 nuclear translocation via reduction of D-gal-induced oxidative stress. Neurodegenerative diseases are treated by targeting the Nrf2 pathway [73]. Keap1, a cysteine-rich protein present in the cytoplasm, binds to Nrf2 under normal conditions. However, when Keap1 denatures, Nrf2 translocates into the nucleus, binds to antioxidant elements (ARE), and turns on antioxidant enzyme genes like SOD and CAT [74]. Studies have shown that aged animals have less nuclear translocation of Nrf2 [67]. This model showed similar results to previous studies, where D-gal treatment inhibited Nrf2 translocation to the nucleus [70], however, treatment of the model with GNL has shown good results.

The neuroprotective effect can be attributed to NQO-1 and HO-1, antioxidant enzymes [75]. In an aged model treated with GNL, the accumulation of nuclear factor Nrf2, increased the expression of NQO-1 and HO-1. Rodent D-gal brain neurotoxicity is also attributed to oxidative stress-induced apoptosis [68]. Herein, the brain hippocampus of mice treated with D-gal showed elevated levels of Bax and caspase-3, and lower expression of Bcl-2 [76]. D-gal-induced aging mice showed that normal neuronal morphology was damaged and expression was increased in the hippocampus. GNL treatment restored cell architecture and morphology and reduced neuronal apoptosis. This indicates that GNL protects hippocampal neurons from D-gal-induced apoptosis. That has been confirmed by histopathological analysis, where GNL reduced the neurodegeneration and necrotic injury and decreased the number of shrunken neurons that were seen in the aging model.

At the pathological level, the current data support a protective role of GNL against D-gal-inducing neurodegeneration in the brain, which agrees with what we have seen in the behavioral and biochemical data. Our results are consistent with the findings of Nam et al. [23] who have recently shown that ascorbic acid ameliorates D-gal-induced impairments through anti-oxidative and anti-inflammatory effects and reduces the pathological changes in the Hippocampus.

^99m^Tc-HMPAO evaluates the regional cerebral blood flow (rCBF) in the brain [77, 78]. As various physiological parameters influence cerebral blood flow, most studies have reported a high diagnostic accuracy for brain perfusion SPECT using ^99m^Tc-HMPAO in dementia [79], Alzheimer’s disease [80], status epilepticus [81], and brain death [82]. Since cerebral blood flow is closely coupled to neuronal physiologic changes, ^99m^Tc-HMPAO has been used as a surrogate marker of neuronal activity changes in certain brain areas where specific hypoperfusion patterns have been observed, for example, in Alzheimer’s disease.

In our study, hypoperfusion in groups II and older groups was observed. Within consistent, several studies reported that most of or specifically the temporoparietal regions were hypoperfused in small animal models and human Alzheimer’s disease [80, 83], and frontotemporal dementia [84]. Aging was reported to be a risk factor for developing cognitive impairment, neurodegeneration, and the subsequent appearance of dementia [85, 86]. Aging also showed reduced ^99m^Tc-HMPAO uptake and cerebral perfusion [87]. This agrees with our study, which reflects the impairment in cognitive impairment. ^99m^Tc-HMPAO images showed that GNL proves to reduce the oxidative stress, neurodegeneration, and cognitive impairment in these experimental groups.

Some studies reported that brain retention of ^99m^Tc-HMPAO reflects an intracellular interaction with glutathione, an antioxidant that comprises most of all free thiols in mammalian cells [88]. Freshly prepared ^99m^Tc-HMPAO forms a lipophilic complex as soon as it is injected intravenously, crosses the brain-blood barrier, and is subsequently trapped intracellularly in a non-diffusible hydrophilic form. This reflects the regional cerebral perfusion. Image analysis has relied on the visual interpretation of ^99m^Tc-HMPAO activity patterns and the statistical analysis of activity ratios within the brain and background ROls. This is a globally well-known image analysis method. Here, ^99m^Tc-HMPAO added to the results and proved strong diagnostic evidence of the effect of GNL in this aging model.

## CONCLUSION

Our data demonstrated for the first time, the antioxidant activity of GNL and its function to attenuate brain hippocampus injury induced *in vivo* by D-gal. In addition, GNL showed to reduce apoptosis and oxidative stress through activation of PI3K/Akt/Nrf2 (Supplementary Figure 3). Consequently, GNL helps in easing cognitive dysfunction and reducing neurological deficits in D-gal-induced aging mouse models. Thus, GNL is a promising therapeutic candidate for age-related diseases.

## Supplementary Materials

Supplementary Methods

Supplementary Figures

Supplementary Table 1

Supplementary Video 1-Group I

Supplementary Video 1-Group II

Supplementary Video 1-Group III

Supplementary Video 1-Group IV

Supplementary Video 1-Group V

Supplementary Video 1-Group VI

Supplementary Video 2-Group I

Supplementary Video 2-Group II

Supplementary Video 2-Group III

Supplementary Video 2-Group IV

Supplementary Video 2-Group V

Supplementary Video 2-Group VI

Supplementary Video 3-Group I

Supplementary Video 3-Group II

Supplementary Video 3-Group III

Supplementary Video 3-Group IV

Supplementary Video 3-Group V

Supplementary Video 3-Group VI
