# Supplementary material for: Geraniol attenuates oxidative stress and neuroinflammation-mediated cognitive impairment in D galactose-induced mouse aging model
Source: Aging (Albany NY). 2024 Mar 20;16(6):5000–26. doi: 10.18632/aging.205677 (PMC11006477; doi:10.18632/aging.205677)
Supplement: Supplementary Methods [file aging-16-205677-s001.pdf]

Supplementary Methods

Isolation of the hippocampus tissue

Retrieve the brain using a spoon and place it on the paper. Using the scalpel blade, remove the cerebellum and approximately one-quarter of the rostral frontal lobes. Run the scalpel blade through the intrahemispheric fissure to completely separate the two hemispheres. Place one hemisphere on the dissecting stage such that the coronal plane of the frontal lobe is facing down. Locate the superior and inferior colliculi on the midbrain; they will be at the "top" of the brain in this orientation. Using the surgical scissors, gently hold the midbrain in place and slide the weighing spatula into the gap between the colliculi and the neocortex. Very gently, continue to slide the spatula down and pull the brainstem, midbrain, and thalamus away, revealing the inside of the lateral ventricle and the medial surface of the hippocampus. Use the sharp edge of the spatula to smoothly sever the fornix, a white fiber bundle located at the anterior/dorsal portion of the hippocampus. With the scissors, gently continue to pull the brainstem, midbrain, and thalamus away without completely severing them from the rest of the brain. The cortex and hippocampus should now lay freely back from the brainstem.
