# Supplementary material for: Geraniol attenuates oxidative stress and neuroinflammation-mediated cognitive impairment in D galactose-induced mouse aging model
Source: Aging (Albany NY). 2024 Mar 20;16(6):5000–26. doi: 10.18632/aging.205677 (PMC11006477; doi:10.18632/aging.205677)
Supplement: Supplementary Figures [file aging-16-205677-s002.pdf]

SUPPLEMENTARY FIGURES

A

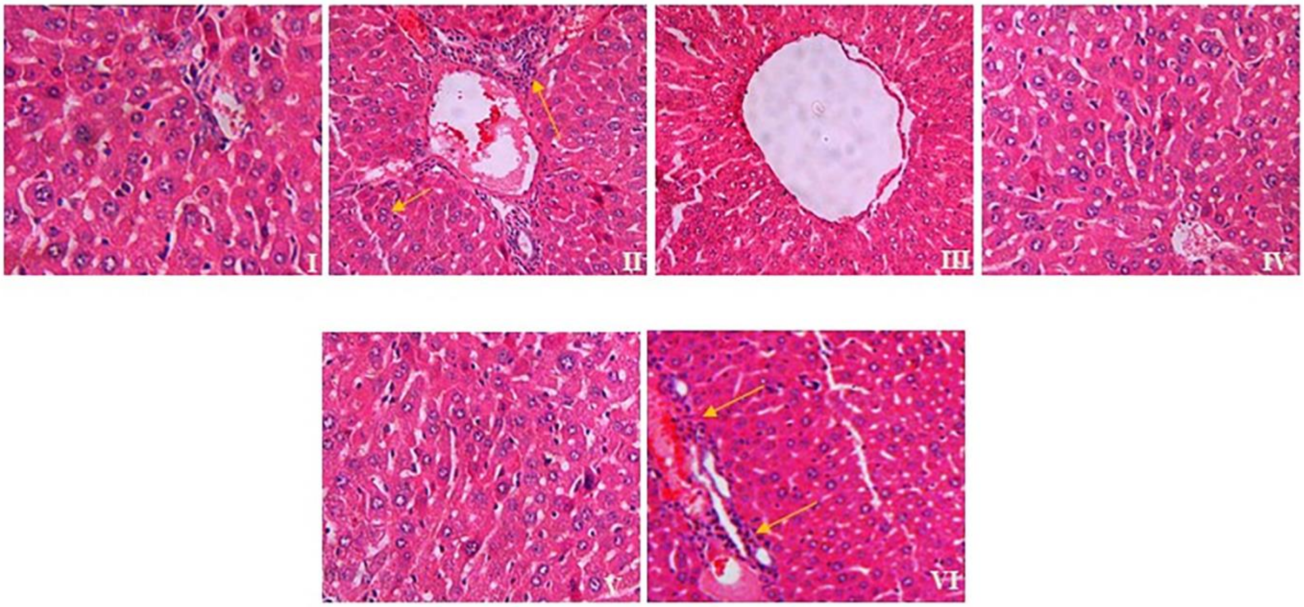

B

Groups	N	Histopathological grade value	
		Hepatocytes necrosis	Inflammatory cell infiltration
**Control**	6	0.0 ± 0	0.0 ± 0
**D-Gal alone**	6	5.37 ± 0.97*	3.21 ± 0.29$^{\#}$
**GNL with D-Gal**	6	2.43 ±0.21$^{\#}$	1.75 ± 0.18$^{\#}$
**GNL**	6	0.0 ± 0	0.0 ± 0
**4 month old**	6	0.0 ±0	0.0 ±0
**16 month old**	6	4.86.37 ± 0.34*	5.34 ± 0.31$^{\#}$

Supplementary Figure 1. The liver of a mouse (H&E, 400×). Staining of liver tissue with H&E after treatment with D-gal alone or with D-gal and GNL together. (**A**) Panel (I) Control hepatocyte cords and nuclei, the central vein, and normal hepatocytes. (II) D-gal alone. Arrows indicate infiltrated inflammatory cells. (III) GNL with D-gal, less sinusoidal congestion, and some infiltrating inflammatory cells. (IV) GNL alone, showing normal architecture. (V) Hepatocyte cords (Hc) of 4-month-old mice, clearly visible nuclei of hepatocytes, central vein, and normal hepatocytes. (VI) Normal-aged mice (16 months); the arrow indicates infiltrated inflammation, just like the mice treated with D-gal only. (**B**) Hepatocyte necrosis and inflammatory cell infiltration into the liver were graded histopathologically (n = 6). Data represent mean ± SD. $^{*}P < 0.05$ vs. control group; $^{\#}P < 0.05$ vs. D-gal-only treated mice. $^{@}P < 0.05$ young vs. old mice.

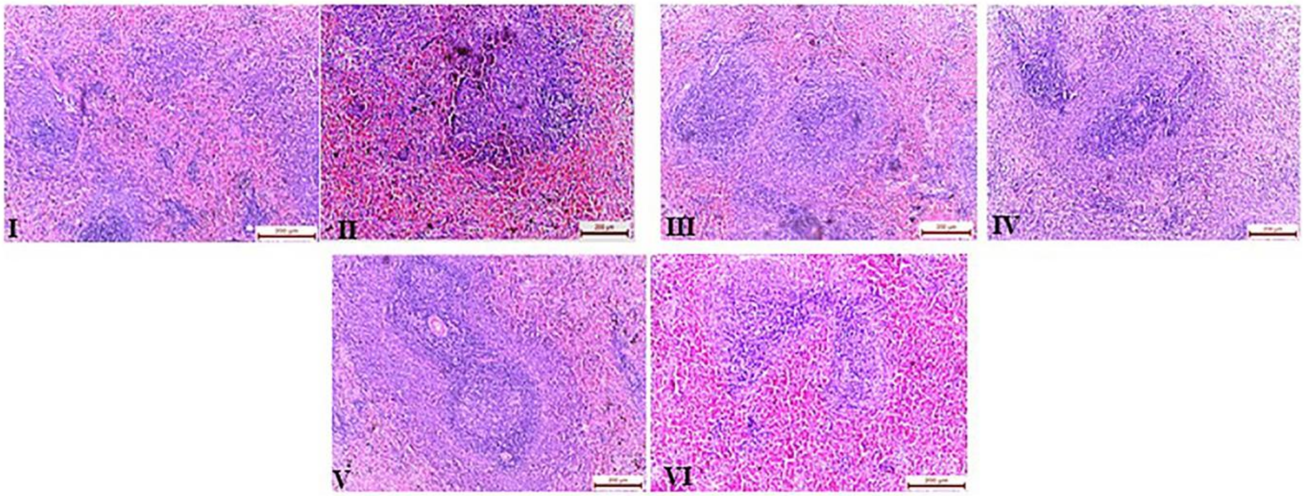

Supplementary Figure 2. Mouse spleen tissue (H&E, 400×). The image shows H&E staining of spleen tissue from mice treated with D-gal alone or with both D-gal and GNL. The H&E-stained section of spleen tissue showed a high degree of morphologic organization in the control group (Group I), D-gal with GNL (III), and GNL alone (IV)-treated animals, while the density of inflammatory cells decreased in both the D-gal-induced aging group (II) and the control (naturally) aging group (VI). Group I: Control; Group II: D-gal alone (150 mg/wt); Group III: D-gal (150 mg/wt) with GNL (40 mg/wt); and Group IV: GNL alone (40 mg/wt). Group V: 4 month-old young animals; Group VI: 16 month-old.

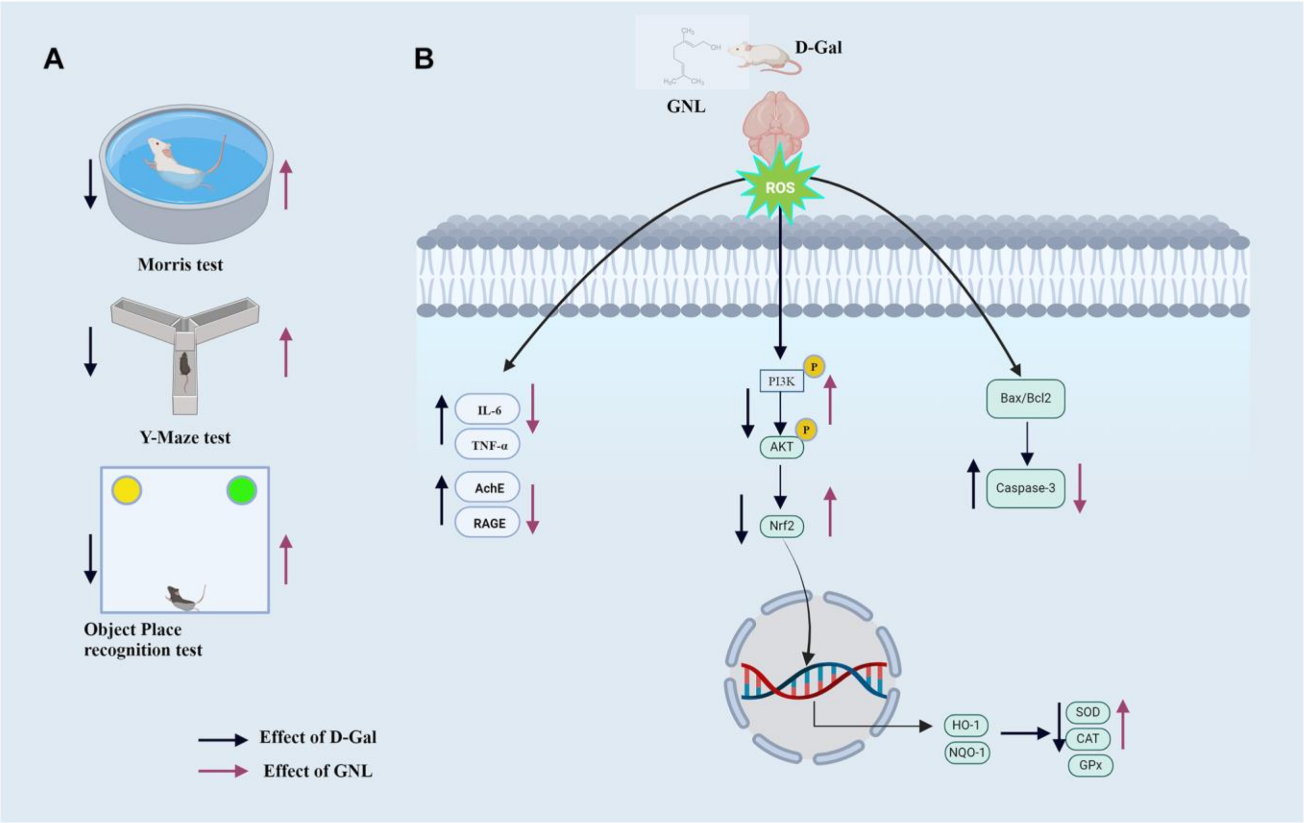

Supplementary Figure 3. Effect of GNL on D-gal induced aging in mice. (**A**) Effect of GNL on aging behavior. (**B**) Effect of GNL on biochemical and molecular mechanism of aging. Image created with https://www.biorender.com/ (dated 03/02/2024).
