# Supplementary material for: Geraniol attenuates oxidative stress and neuroinflammation-mediated cognitive impairment in D galactose-induced mouse aging model
Source: Aging (Albany NY). 2024 Mar 20;16(6):5000–26. doi: 10.18632/aging.205677 (PMC11006477; doi:10.18632/aging.205677)
Supplement: Supplementary Table 1 [file aging-16-205677-s003.pdf]

SUPPLEMENTARY TABLE

Supplementary Table 1. Antibody details of the proteins used in immunoblot analysis.

Antibody	Molecular weight (kDa)	Dilution
BACE-1	70	1:2000
HO-1	32	1:5000
γ-GCLC	73	1:5000
RAGE	58	1:2000
pPI3K	85	1.2000
PI3K	85	1:2000
pAKT	60	1:5000
AKt	60	1:2000
Nrf2	62	1:5000
Bcl-2	28	1:1000
Bax	20	1:1000
Cleaved Caspase-3	17	1: 1000
Secondary antibodies goat anti-rabbit	–	1:10,000
Secondary goat anti-mouse	–	1:10,000
β-actin	43	1:10,000
